# Pain Sensitivity and Observer Perception of Pain in Individuals with Autistic Spectrum Disorder

**DOI:** 10.1155/2013/916178

**Published:** 2013-06-13

**Authors:** C. S. Allely

**Affiliations:** Institute of Health and Wellbeing, University of Glasgow, RHSC Yorkhill, Glasgow G3 8SJ, UK

## Abstract

The peer-reviewed literature investigating the relationship between pain expression and perception of pain in individuals with ASD is sparse. The aim of the present systematic PRIMSA review was twofold: first, to see what evidence there is for the widely held belief that individuals with ASD are insensitive to pain or have a high pain threshold in the peer-reviewed literature and, second, to examine whether individuals with ASD react or express pain differently. Fifteen studies investigating pain in individuals with ASD were identified. The case studies all reported pain insensitivity in individuals with ASD. However, the majority of the ten experimental studies reviewed indicate that the idea that individuals with ASD are pain insensitive needs to be challenged. The findings also highlight the strong possibility that not all children with ASD express their physical discomfort in the same way as a neurotypical child would (i.e., cry, moan, seek comfort, etc.) which may lead caregivers and the medical profession to interpret this as pain insensitivity or incorrectly lead them to believe that the child is in no pain. These results have important implications for the assessment and management of pain in children with ASD.

## 1. Introduction

Autistic Spectrum Disorder (ASD) describes a range of conditions classified as pervasive developmental disorders (PDDs) in the Diagnostic and Statistical Manual of Mental Disorders (DSM). PDDS include autism, Asperger syndrome, pervasive developmental disorder not otherwise specified (PDD-NOS), childhood disintegrative disorder, and Rett syndrome. However, typically only the first three are considered part of the autism spectrum [1]. ASDs have an onset in early childhood and adverse, often lifelong, effects on communication, socialisation including tendencies toward restricted interests and/or repetitive behaviours [2]. Often associated with these symptoms are sensory-perceptual anomalies which occur in approximately 70% of cases [3]. To determine the prevalence of autism and related disorders, the Centers for Disease Control (CDC) conducted a study examining 8-year-old children living in 14 sites in the United States and found that 1 in 150 children are living with an ASD [4]. Despite the numerous studies attempting to clarify the pathogenesis of ASD, the causes remain uncertain [5].

Individuals with ASD may experience sensory abnormalities related to sight, hearing, touch, smell, and/or taste that include an increased sensitivity to pain. The processing of these types of incoming information might be distorted; rain might sound like gunfire, clothing might feel like sandpaper, or fingers shampooing a scalp might feel like sharp metal [6]. Such unusual responsiveness to the environment has been suggested to be partly due to stimulus overselectivity, the tendency of individuals with ASD to respond only to a very limited amount of the relevant sensory information [7]. Both hyposensitivity and hypersensitivity are exhibited in the same individual [3, 8–10]. Stereotyped and self-injurious behaviours (SIBs) are also exhibited in significant numbers of individuals with ASD [11–15] which has been associated with apparent pain insensitivity [16]. As many as 70% of ASD patients may show self-injurious behaviour at some point in their lives, but this is typically found in more severely affected individuals and takes on many forms including head banging, scratching, bruising, and biting [17]. However, the role of pain in relation to self-injury is unclear [18–22].

Numerous biochemical theories have been put forward to explain the apparent pain insensitivities in individuals with ASD. Certain repetitious activities such as rocking, arm flapping, or pacing produce an increase (or build up) of the level of released endorphins which can lead to a reduction of the sensation of pain, which may explain why children with an ASD who have physical accidents report feeling less pain when the accidents take place later in the day [23]. A number of researchers have also suggested that excessive brain opioid activity could explain the apparent pain insensitivity of ASD and contribute to or even determine the pathogenesis of ASD [24–30]. The opioid hypothesis for ASD postulates that this “hyperfunction of the endogenous opioid system” may actually explain some, if not all, of the symptoms associated with ASD including (1) reduced socialisation (and aloofness), (2) reduced clinging in animals, (3) diminished crying, (4) repetitive stereotyped behaviours, (5) promotion of convulsive activity, (6) insensitivity to pain, (7) episodes of motor hyperactivity alternating with hypoactivity, and (8) affective lability [27].

To date, relatively little research on the sensitivity to painful stimuli, or the expression of pain, in infants, children or adults with ASD has been conducted. Accurate pain assessment, in order to provide appropriate and timely care, can be a challenging task especially in children with ASD [31]. However, pain assessment strategies for children with ASD are poorly understood [32] and relatively little is written about the relationship between pain and ASD in the pain literature [33]. Due to communication and assessment difficulties, there is a greater likelihood that their pain may go unrecognised and untreated (e.g., [34, 35]). Another potential barrier to assessing pain in children with ASD is the prevailing belief, frequently based on anecdotal observation or clinical impression, that pain insensitivity is a common feature in children with ASD (e.g., [2, 36–40]). Parents, caregivers, and mental health professionals have reported that some children with ASD appear to withstand painful stimuli (bumps, cuts, etc.) show absence of nociceptive reflexes (e.g., absence of hand withdrawal reflex when burning oneself), or lack of protective body position in cases of broken legs or arms [41]. However, nearly all of the support for this notion of pain insensitivity is derived from anecdotal reports and limited clinical observations [24–29, 42–44]. Despite the lack of systematic studies of pain sensitivity and reactivity in ASD, the presence of pain insensitivity in ASD has been given further validation because of its inclusion as an associated feature in standard diagnostic texts. In DSM-IV and DSM-IV-TR “a high threshold for pain” is described [2, 45] while in DSM-III the “ignoring of pain” is described (APA, 1987). Not only are children with ASD considered to have “reduced pain sensitivity,” but they have also been described as “not feeling pain as intensely as others” [27], having an “indifference to pain” [44] and having a “high threshold for pain” [2]. The belief that children with ASD are insensitive to pain may bias observers' judgements of pain in these children [46].

It is important to understand the behaviours observers can use to assess pain in children and adults with ASD and to understand the potential bias of pain sensitivity information on observers' judgements of pain. Over the last decade there has been a plethora of studies investigating pain expression and perception in individuals with intellectual disabilities or individuals with developmental disabilities (often the exact diagnostic nature of these groups is not specified) (i.e., [47–50]). By contrast, peer-reviewed literature investigating the relationship between pain expression and perception of pain in individuals with ASD is sparse. Research on pain in children with developmental disabilities has almost exclusively relied on observational or behavioural assessment measures [51, 52]. The present systematic review was carried out using PRISMA guidelines [53] to primarily identify and examine the evidence for the widely held belief that individuals with ASD are insensitive to pain or have a high pain threshold. Additionally, this review will examine whether individuals with ASD react or express pain differently.

## 2. Method

Internet-based bibliographic databases (PsycINFO and PubMed) were searched to access studies which examined pain in individuals with ASD only (authors reported no comorbidity in their sample). Studies which investigated pain in individuals with ASD who also had another disorder, such as mental retardation, were not included because the present review was specifically interested in the impact *pure* ASD has on pain sensitivity and expression of pain. Studies which investigated pain in individuals with ASD on a psychological/behavioural level were included. Numerous studies were excluded as they explored more medical issues related to ASD such as gastrointestinal symptoms (i.e., bowel inflammation and alterations in intestinal microflora). 

**Figure 1:**
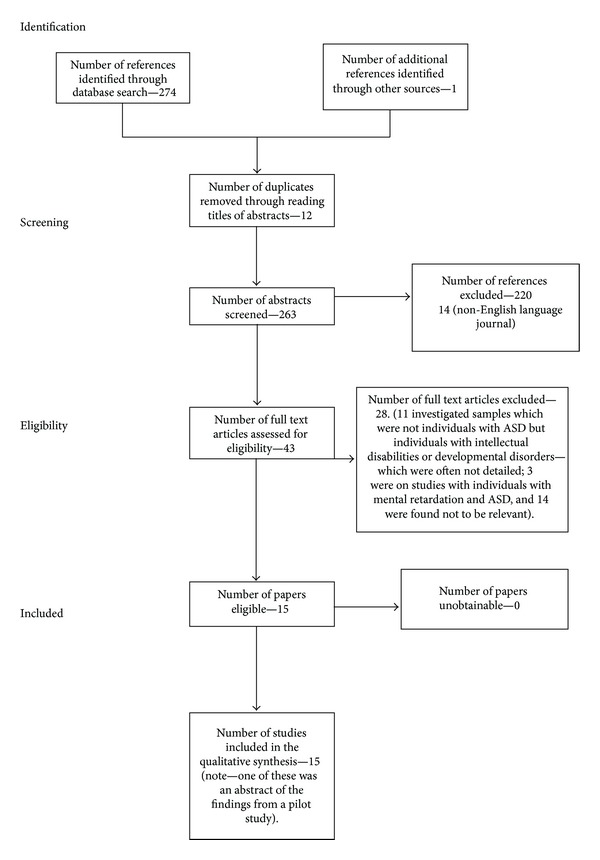
Flow of information through Systematic Review.

It is commonly reported in the literature that sensory disturbances can feel painful to individuals with ASD. For instance, rain might sound like gunfire and the individual finds this so painful that they have to cover their ears. However, studies which discussed these types of painful experiences were not included as the present review is interested in what is considered to be well-known painful events to the majority of individuals, such as needles in the skin and high and cold temperature. The process of eliminating nonrelevant papers can be seen in the flowchart (following PRISMA guidelines, [53]) (Please see Figure 1 for the Flowchart). Duplicates were excluded prior to the retrieval of references. Searches on the two databases were originally conducted on October, 18, 2012 and updated on February, 6, 2013. The following search criteria were entered into PubMed: [“pain” (title/abstract) AND “autis∗” (title/abstract)] which returned a total of 126 references. The following search criteria were entered into PsycINFO: [“pain” (all text) AND “autis∗”] which returned a total of 148 references. Combining the abstracts returned on these two databases, there was a total of 274 abstracts. In addition to these database searches, numerous permutations of ASD and pain were entered into Google Scholar and thoroughly searched for any additional articles not found in the database searches. For instance, [Asperger AND pain]; [autism AND pain]; [ASD and pain]. These searches only returned one additional article which was an abstract of a pilot study (the findings of which have not since been published in a peer-reviewed article to the authors knowledge). 

Abstracts for each reference were obtained and screened using the following criteria.

*Inclusion Criteria*

1. Human study population.
2. Investigated psychological/behavioural pain response in individuals with ASD.

*Exclusion Criteria*

1. Paper not published in English.
2. Dissertations.
3. Book reviews.
4. Studies which investigated a sample that comprised of individuals with a disorder other than ASD (for instance, developmental disabilities or intellectual disabilities) or ASD with a comorbidity (i.e., ASD and mental retardation) were excluded.

*Screening*. In the first stage, papers were rejected which

- investigated medical issues (such as intestinal inflammation) or clinical psychopharmacology aspects of ASD,
- were not published in the English language.

For the next stage, papers were rejected which were not studies that involved a sample of individuals with ASD.

In addition, review papers and book chapters which were clearly reviews were excluded. Full documents were obtained for the remaining records.

## 3. Results

Five case studies and ten experimental studies were found in the PRISMA search that investigated some aspect of pain in individuals with ASD. 

### 3.1. Case Studies

Table 1 details the five case report studies which explored pain perception, expression, or observer perception of pain in individuals with ASD. 

Pain experts might be underrecognising signs and symptoms of ASD in their patients, a notion which led Bursch et al. [33] to explore this in two patients (Tony and Gregg) who displayed signs and symptoms indicative of possible ASD. Tony's mother reported a possible sensory disturbance in early childhood in that he liked to belt his trousers extremely tightly, which most children would have found painful, but Tony liked the sensation. His mother reported that he once grabbed a hot frying pan and did not seem to respond in a way typical of someone in pain. Despite both adolescents showing obvious signs and symptoms indicative of an ASD, review of previous medical records and parental interviews suggested that health care professionals did not identify the need for evaluation of these unusual characteristics. This is clinically useful to recognise because any type of pain might be exacerbated by sensory processing abnormalities and/or persistent arousal that often characterise patients with ASD [54, 55]. 

 Elwin et al.[56] reviewed 17 works published in English or Swedish and 10 autobiographies to explore hyper- and hyposensitivity in individuals with ASD in the context of verbal expression. The autobiographies were written by individuals who all had an ASD diagnosis. The authors found much evidence to suggest pain insensitivity (hyposensitivity) in individuals with ASD in that pain could be indistinctly experienced. Several authors indicated that they had a very high pain threshold. For instance, one individual reported “Injuries could easily go undetected. In there, I was given a punch in the stomach, every day, though usually only one. Perhaps I was not much fun to hit because I had a very high pain threshold, and even when I did hurt I never showed what I felt. I did not know that was what you should do” [57, page 92]. They also found evidence of pain sensitivity (hypersensitivity). However, for the purposes of the present review we have excluded this as from the descriptions given in the paper by Elwin et al. [56], this was pain which was experienced as a result of sensory abonormalities not specifically related to what would typically be defined as a painful event (such as bodily injury or needle injection). They reported cases where the individual reported feeling excruciating pain racking through her head in response to a fog horn.

Rutherford [58] described the development of an infant (N.F.) who was later diagnosed with ASD in direct comparison to the development of his twin, from the prenatal period to the age of four years, through the examination of personal journals and medical records kept by the mother of the twins. Several differences in development between the twins, some as early as six months of age, were found and of particular interest was the observation that N.F. frequently showed insensitivity to pain which was exhibited as early as six months of age. Differences reemerged by the age of four years, at which point N.F. would wake up frequently during the night, sometimes as many as nine times. He would cry and yell during these times and his mother thought that he appeared to be in extreme pain.

Autoextraction of teeth (self-extraction of a tooth) is an unusual form of self-injurious behaviour (SIB) and is rarely seen in children with ASD. Ross-Russell and Sloan [17] present the case of a seven-year-old boy with mild ASD who experienced unexplained dental pain and subsequently went on to extract his own lower right deciduous canine tooth. He has also demonstrated SIB in the form of head banging. He was brought to the clinic complaining of pain of about one week's duration that was increasing in intensity, was present most of the time, and was not sensitive to hot, cold, or sweet stimulus. Within 24 hours the patient was again back at the clinic as the pain had not resolved with ibuprofen and by this time his lower right deciduous canine was very slightly mobile. The tooth had normal root anatomy and no evidence of alveolar bone loss was evident but by the next day the patient had extracted his lower right deciduous canine tooth, which was witnessed by his mother. He claimed it had been “itching” him until he got it out. This case indicates that the patient was insensitive to pain to some degree. 

 Mieres et al. [32] describe how a nurse and a physical therapist in an interprofessional (IP) school-based clinic worked together to meet the needs of a nine-year-old child with pervasive developmental disorder, not otherwise specified, with atypical classroom behaviours and declining student performance. The child denied pain of any type, using a 0- to 10-point visual analogue scale. The IP team noted that the student was speaking less to other students, faculty, and staff. Although such behaviour could be a sign of withdrawal, it may also be a sign of oral problems. As five weeks had passed without any change in the student's demeanor, the child was referred to a dentist who discovered a severe abscess affecting two teeth. The dentist reported that an abscess of this size without pain is unusual, given the size, the depth, and the proximity to bone. The abscess required two 10-day rounds of antibiotics until the infection was completely remedied. Two months later, the same student arrived in the clinic and stated that he was “not able to concentrate and the noise was bothering him.” Again, the student denied pain, using a 0- to 10-point pain scale, both verbal and pictorial. When given the same scale of 0 to 10 and instead asked, “How uncomfortable are you?” the student stated about a 7. Multiple superficial cuts and lacerations, some covered with bandaids, were seen bilaterally. When the mother was called from the clinic, she stated that over the weekend the student played in the sand dunes without shoes. She further stated that the other children playing with him left quickly after complaints of painful pinching in their legs and feet. The student continued to play in the dunes without any sign of discomfort and could not understand why the other children were complaining. Later, the mother discovered bleeding cuts on his feet and he was taken to a 24-hour clinic, where the physician removed 14 sand spurs. The student helped in the removal of the sand spurs without complaints of pain even when extractions were deep. However, the following morning, he complained of excessive noise, did not wish to be touched, and covered his head. The student stated at first that he had a 0 in a pain scale of 0 to 10. However, when asked, “How uncomfortable are you?” the student indicated a 7. For this student, pain was an unreliable indicator of both a dental infection and piercing of skin by thorny objects, preventing timely treatment. 

In sum, the five reported case studies all seem to provide some support to the widely held belief that individuals with ASD are insensitive to pain or have a high pain threshold.

### 3.2. Experimental Studies

Table 2 details the experimental studies which explored pain perception, expression, or observer perception of pain in individuals with ASD. There were ten experimental studies identified in the search of which one was an abstract presenting the findings from a pilot study. The ten studies are split up into five different sections under the subheadings of “Facial Activity to Pain Stimuli in ASD” (no. 3); “Pain Sensitivity Experienced in ASD” (no. 4); “Embodied Pain in ASD” (no. 1); “Relationship between Opioid Hormone and ASD” (no. 1) and “Oversensitivity to Pain and Age of Diagnosis of ASD” (no. 1). 

### 3.3. Facial Activity to Pain Stimuli in ASD

 Facial activity has been found to be a major determinant of observers' judgements of pain in infants [59], children [60], and adults with cognitive impairments [61]. The widely held belief that children with ASD are less sensitive to pain compared to neurotypical children may bias observers' interpretation of pain expression/behavioural reactivity in these children. Messmer et al. [46] investigated whether the perceptions of pain in children with ASD could potentially be influenced by the belief that children with ASD are insensitive to pain. Twenty-seven undergraduate psychology students who had no previous experience with children with ASD were recruited at the University of British Columbia. The sample consisted of 23 females and four males, with a mean age of 20.11 years. Nineteen of the participants identified themselves as Caucasian, seven participants identified themselves as Asian, and one identified him/herself as “other.” Observers received information that pain experience in children with ASD is either the same as, more intense than, or less intense than children without ASD. After viewing six video clips (which were obtained from a previous study by Nader et al. [62], described below) of children with ASD undergoing venepuncture, observers estimated pain intensity using a visual analogue scale. Venepuncture is a medical procedure which requires the use of a needle to puncture a patient's vein. Puncturing a vein provides direct access that allows for extraction of venous blood or insertion of medication or fluids directly into the blood stream and is the same basic procedure which is used to extract blood for blood donations. The sample of children with ASD used for the current study consisted of four boys and two girls between the age of three and seven. The clips consisted of the 10 seconds immediately preceding the injection and the 10 seconds immediately after needle insertion. Participants were randomly assigned to one of the three groups. Group A consisted of seven participants, and groups B and C consisted of 10 participants. Each group read a two-page booklet with information taken from “Children with Autism: A Parent's Guide Describing Features of Autism” [63]. Within the general account was a description of the pain experience of children with ASD. This description of pain in individuals with ASD was different for each group, saying either (a) “Children with autism appear to respond to pain in the same way that children without autism do”; (b) “Children with autism also appear to respond to pain differently than children without autism. In particular, they feel pain more than other children. This has been termed “pain hypersensitivity” and has recently been documented in research on children with autism”; or (c) “Children with autism also appear to respond to pain differently than children without autism. In particular, they seem to have a high tolerance for pain and do not appear to feel pain as much as other children”. 

After reading the booklet, participants watched six video clips of children with ASD undergoing venepuncture. The video clips had been previously coded for facial activity using the Child Facial Coding System (Child Facial Action Coding System Revised Manual, CFCS [64]). The CFCS is a facial coding system which was created as a way to assess pain experiences in children. Thirteen explicitly defined facial actions (e.g., brow lower, eye squeeze, and nose wrinkle) are coded, in terms of frequency and intensity, by a trained CFCS coder using stop-frame and slow-motion video editing equipment. After each video clip, participants rated the pain intensity of the child on a visual analogue scale (VAS), a 100 mm horizontal line anchored on the left by “no pain” and on the right by “worst possible pain.” Participants placed a mark on the line to indicate how much pain they thought the child was feeling. The VAS is a valid measure for assessing pain intensity [65]. Mean pain intensity scores on the VAS were compared to the average facial pain activity scores from the CFCS. A Spearman rank order correlation suggested that the order of VAS ratings was highly correlated with the order of the CFCS scores (*rs* = 0.943, *P* < 0.01). In sum, the main findings of this study by Messmer et al. [46] were that children who received lower scores on the CFCS were judged to be experiencing a lower intensity of pain and children who received higher scores on the CFCS were judged to be experiencing a higher intensity of pain. Thus, Messmer et al. [46] found that observers' ratings of pain in children with ASD were* not* influenced by information regarding the pain experience in children with ASD and that they were able to use facial activity as one basis for estimating pain in children with ASD. This study also indicates that the children's experience of pain is communicated, at least to some degree, through their facial activity.

Nader et al. [62] conducted a study in order to examine the behavioural response of children with ASD during venepuncture using an objective observational measure of pain and distress. In addition to this objective measure, they also examined parents' assessments of pain behaviour in children with and without ASD, including comparison of the relationship of parental reports with behavioural measures. All of these measures were compared to the same assessment conducted on control children during the same procedure. Nader et al. [62] recorded behavioural distress and facial reactions of pain in 21 three- to seven-year-old children with ASD and 22 nonimpaired children during venepuncture. Parents provided observer reports of pain and facial activity was used as an objective behavioural measure of pain. Detailed coding of videotapes were performed using the Child Facial Coding System [64] (which was the objective measure also used in the study above by Messmer et al. [46]). An objective measure of distress was also used in the present study, namely, the Observational Scale of Behavioral Distress (OSBD). The OSBD [66] is a coding system designed to assess behavioural distress in children undergoing painful medical procedures. OSBD consists of eight operationally defined behaviours indicative of anxiety and/or pain behaviour in children. 

 Observer reports of pain from the parents were measured using the following two procedures. Histories of pain sensitivity were assessed by asking the parents to report on prior pain reactions of their children using the Non-Communicating Children's Pain Checklist (NCCPC) [67]. Parents were also asked to provide a summary report of their child's pain temperament by responding to the following statement: “My child is very sensitive to pain of bumps or cuts or other common hurts.” The parent responded to this question on a scale of 1 = not typical/characteristic to 5 = very typical/characteristic. Lastly, the Faces Pain Scale (FPS; [68]) was given to the parents. This consists of seven faces showing gradual increases in pain expression from left to right (neutral to pain). The parents were asked to select the face that they felt represents the degree of pain experienced by their child during the venepuncture procedure. 

Findings from the study by Nadar et al. [62] revealed that the behavioural responses of the children with ASD were overall similar to the comparison group, except the substantial facial pain reactivity instigated by the venepuncture in the children with ASD exceeded that found in the control group. The degree of concordance between parental report and observed pain responses were consistently better for the comparison group. For the ASD group, no significant correlation was observed between the FPS scores provided by the parents and the facial pain responses of the children, *r* = −0.154, *P* > 0.05. Interestingly, children with ASD who had been assessed by their parents as having a lower pain sensitivity and reactivity tended to show greater facial reactions and behavioural distress in response to the venepuncture. Using FPS scores as a measure of parental assessment of pain response following the venepuncture, parents of children with ASD reported observing more pain in their children during the venepuncture (*M* = 4.29, SD = 1.45) compared with parents of the children without ASD (*M* = 2.75, SD = 1.90; *t*(41) = 2.97, *P* < 0.05). Using the NCCPC as a retrospective measure of parental assessment of typical pain reactivity in their children, scores did not differ between the ASD group (*M* = 60.33, SD = 13.50) and comparison group (*M* = 58.41, SD = 14.19; *t*(41) = 0.46, *P* > 0.05). Parent reports of pain temperament in children with ASD (*M* = 2.72, SD = 1.32) were similar to parent reports of pain temperament in the children without ASD (*M* = 2.82, SD = 1.30; *t*(38) = −0.23, *P* > 0.05). In addition, although the ASD severity of the ASD group was well characterised and ranged from mild to severe, there was no information about level of intellectual functioning for this group. Overall, these findings demonstrate that children with ASD can display a significant behavioural reaction in response to a painful stimulus which is in contrast to the widely held belief in the literature that individuals with ASD are insensitive to pain. However, the study also shows that some of the caregivers did not interpret their child's pain expression accurately. Children with ASD, who had been assessed by their parents as having a lower pain sensitivity and reactivity, tended to show greater facial reactions and behavioural distress in response to the venepuncture. However, this is difficult to draw strong conclusions from this since it may be that the event was simply more distressing for the individuals with ASD rather than that they had any greater degree of pain sensitivity. 

In another study, Tordjman et al. [41] examined behavioural and physiological pain responses, plasma beta-endorphin levels and their relationship in 73 children and adolescents with autism and 115 normal individuals matched for age, sex, and pubertal stage during blood drawing. Pain reactivity was assessed for patients in three different observational situations. (1) in day care, where two caregivers independently rated overall pain reactivity on a daily basis during the month preceding the blood drawing; (2) at home, where parents rated pain-related behaviour during the same month as the caregivers. In this situation, there were enough daily life situations involving pain to distinguish reactions to a variety of types of noxious and painful stimuli such as being burned, having internal pain (tooth pain, ear infection, headache, etc.), and other accidental painful stimuli (cutting, pinching, banging, etc.); (3) during the blood drawing at a medical centre, when a direct clinical observation was conducted by a nurse and child psychiatrist not belonging to the caregiver team. Normal controls were similarly assessed for pain reactivity to the venepuncture using the Pre-Linguistic Behavioral Pain Reactivity Scale (PL-BPRS) [69]. The scale looks at five different pain scenarios, namely, (1) paradoxical pain reactivity, the apparent pleasure reaction to a painful stimulus (such as smiling or laughing); (2) absence of pain reactivity, absence of nociceptive reflexes (such as absence of hand withdrawal reflex when burning oneself or absence of arm withdrawal reflex from the needle during a blood drawing); (3) hyporeactivity to pain, incomplete pain reactivity or abnormally delayed reaction time to painful stimulus; (4) normal pain reactivity such as cries, screams, moaning, grimaces, reflexes of nociceptive withdrawal, lack of movement, body orientation, and glance towards the painful area, and lastly, (5) hyperreactivity to pain, disproportionate cries, and screams given the painful stimulus (with hypersensitive light touch). A checklist was used to indicate the presence or absence of SIB, aggressive behaviours directed against others, stereotyped behaviours, and social withdrawal during the blood drawing situation. Physiological measures included plasma b-endorphin levels analysis and a heart rate measurement to examine cardiovascular response to the blood drawing (with a stethoscope placed on the thorax considering that some patients can react negatively when their wrist is touched) immediately before and after the venepuncture (15-second measurement period). 

Tordjman et al. [41] found that across the three observational situations, abnormal behavioural responses to painful stimuli were highly prevalent in individuals with ASD of low to moderate functioning. In general, there was a shift to hyporeactive or absent pain reactions in the ASD group. A high proportion of individuals with ASD displayed absent or reduced behavioural pain reactivity at home (68.6%), at day care (34.2%) and during venepuncture (55.6%). Although this pattern of observed behaviour is consistent with a number of previous studies, most prior reports did not distinguish pain reactivity from pain sensitivity. It is critical to keep this distinction in mind and not to conclude that absence of behavioural pain reactivity means absence of pain sensitivity. Despite their high rate of absent behavioural pain reactivity during venepuncture (41.3% versus 8.7% of controls, *P* < 0.0001), individuals with ASD displayed a significantly increased heart rate in response to venepuncture (*P* < 0.05). This response (Delta heart rate) was significantly greater than for controls (mean ± SEM; 6.4 ± 2.5 versus 1.3 ± 0.8 beats/min, *P* < 0.05). This strongly indicates that prior reports of reduced pain sensitivity in ASD are related to a different mode of pain* expression* rather than to an insensitivity or endogenous analgesia. Plasma beta-endorphin levels were higher in the ASD group (*P* < 0.001) and were positively associated with ASD severity (*P* < 0.001) and heart rate before or after venepuncture (*P* < 0.05), but not with behavioural pain reactivity. This is inconsistent with the opioid theory of ASD that would suggest that high levels of plasma beta-endorphin is associated with behavioural pain reactivity. In addition to the physiological response to the venepuncture, behavioural changes following the venepuncture or other painful stimuli occurring at home and day hospital (SIB, aggressive behaviours, stereotyped behaviours, social withdrawal) also suggest that children with ASD perceive pain, but do not express it in the same way that control children do. 

The findings by Tordjman et al. [41] also show that a significant proportion of individuals with ASD did not display low/absent overall pain reactivity according to the parental, caregiver, and blood drawing evaluations. In fact, the majority (78%) of individuals with ASD were actually found to exhibit normal behavioural reactivity to burning; highlighting the importance of distinguishing different types of painful stimuli. Lastly, 22% of individuals with ASD displayed normal behavioural pain reactivity to the venepuncture and 15.9% displayed hyperreactivity which is in agreement with Nader et al. [62]. In sum, this study indicates that there may be different subgroups within the ASD population. One subgroup may experience pain insensitivity, another pain sensitivity, and the other normal pain sensitivity. However, there are numerous factors to consider when making such a conclusion at this early stage and this is outlined in the discussion. For instance, this present study found that the majority of individuals with ASD exhibited normal pain reactivity to burning. It may be that individuals with ASD may need to experience a particular high level of pain such as burning before they express normal pain reactivity. However, when the painful event is not so severe some individuals with ASD may have difficulty in expressing the pain. 

### 3.4. Pain Sensitivity Experienced in ASD

Klintwall et al. [70] investigated sensory abnormalities in a population-based group of 208 20-54-month-old children, diagnosed with ASD and referred to a specialised habilitation centre for early intervention. Children were subgrouped (eight in total) based upon degree of autistic symptoms and cognitive level by a research team at the centre. Parents were interviewed systematically about any abnormal sensory reactions in the child. In the whole group, pain and hearing were the most commonly affected modalities. An interview according to the PARIS schedule (developed by Gillberg and colleagues within the “Paris Autism Research In Sib-pairs” study, [71]) was performed with one of the parents. This interview included structured questions about the child's sensory reactions to light, sound, smell, and so forth. However, for the purposes of this review their results for underreactivity to pain, underreactivity to heat, and underreactivity to cold are reported. Only clinically significant sensory abnormalities were scored as “present” in the study. Children in the most typical ASD subgroup (nuclear autism with no learning disability) had the highest number of affected modalities. There were no group differences in number of affected sensory modalities between groups of different cognitive levels or level of expressive speech, supporting the notion that sensory abnormality is very common in young children with ASD and providing further justification for inclusion of this symptom in the diagnostic criteria for ASD in the upcoming DSM-V. From the total group of 208 children, at least one type of major sensory abnormality was registered in 158 individuals (76%). The most commonly reported sensory abnormality was overreactivity to sound (44%) and underreactivity to pain (40%). Underreactivity to cold and heat was reported for 22% and 7%, respectively. Interestingly, children with self-injurious behaviours had a greater number of affected sensory abnormalities (*M* = 2.0, SD = 1.5, *n* = 61) compared to children with no such self-injurious behaviours (*M* = 1.3, SD = 1.2, *n* = 147); *t*(206) = 2.791, *P* = 0.006. Therefore, this study provides some support to the widely held belief that many individuals (40%) with ASD are insensitive (under reactive) to pain. 

Cascio et al. [72] recruited eight adults with high-functioning ASD (clinical diagnoses of either Autistic Disorder or Asperger Disorder; DSM-IVTR; [2]); there were seven males and one female (mean age 29.3 years, range 20–45). Eight adults without ASD were recruited from the community, selected to match each individual with autism on age and gender (mean age 29.0 years, range 21–45). Each participant completed a brief questionnaire, the Adult Sensory Profile [73] to determine whether groups differed in terms of their experience with sensory stimuli in everyday life. Cascio et al. [72] compared tactile sensation in adults with ASD compared to controls on two sites of the body: (1) the hairy skin of the right dorsal forearm and (2) the glabrous skin of the right thenar palm. A variety of tactile sensations were investigated. However, for the purposes of this review only those that were pain related are reported here. These were the thermal sensation—cold pain and heat pain. Participants were instructed to respond as soon as the stimulation reached a point of being “painfully or uncomfortably hot (or cold).” In order to alleviate any anxiety about the pain stimuli, participants were reminded that the device was limited to temperatures that are too mild to produce skin damage, and that their response triggered the return of the thermode to its baseline temperature. For cold pain, there was a main effect of site (*F* = 5.12, *P* = 0.0250), and group (*F* = 3.84, *P* = 0.0518), with the ASD group displaying average cold pain thresholds of 16.68°C, compared to the control group average of 9.04°C. For heat pain, there was a significant effect of group (*F* = 6.79, *P* < 0.01), and site (*F* = 7.37, *P* = 0.0073), and a significant group *x* session interaction (*F* = 8.18, *P* = 0.0048). The average threshold for the ASD group was 43.66°C, while that of the control group was 46.58°C. Overall, the ASD group showed a greater degree of pain sensitivity to thermal pain at both sites recorded in this study as this group's cold and heat pain thresholds were lower compared to the control group.

Bandstra et al. [74] examined self-reported and parent-reported pain in 20 high-functioning youths with ASD (17 boys; 3 girls) and 20 typically developing controls (16 boys; 4 girls) ranging in age from 8 to 18 years and matched on age and IQ. This is the first study to assess the self-report of pain, using vignettes, in high-functioning children and adolescents with ASD. The Charleston Pediatric Pain Pictures (CPPP) are a series of 17 cartoon pictures depicting scenes of medical, play, and home situations [75]. Each drawing has a central figure of a young non-sex-specific child lacking facial expression, who is engaged in an activity. Thirteen of the 17 scenarios depict pain-provoking events and each has a short verbal vignette that describes the event taking place in the picture. One example of the 13 pain scenarios was: “You touched the hot stove and burned your hand. Show me how much hurt you would have”. The amount of pain the participants would expect to feel was self-reported using the Faces Pain Scale-Revised (FPS, [76] and a Numeric Rating Scale (NRS) in a series of validated hypothetical pain situations depicted in cartooned images (e.g., scraping knee on pavement). The FPS-R is comprised of 5 line drawings of faces, presented horizontally, representing increasing levels of pain, typically from no pain (0) to extreme pain (10). In addition to the FPS-R, participants and their parents were asked to rate the pain of the hypothetical situations using an NRS. The NRS was provided using a 0 to 5 scale not only to ensure simplicity of the task for children in the study, but also to provide participants with the same number of response options as provided in the FPS-R. So children and adolescents were asked to provide ratings of their hypothetical pain using both the FPS-R and the NRS, whereas parents were asked to only provide ratings of their child's pain using the NRS. Findings revealed no differences between the pain ratings of youths with ASD or their parents as compared with a sample of typically developing youths. 

The lack of differences in pain intensity ratings between the ASD and control youths in the study by Bandstra et al. [74] conflicts with other recent findings which showed greater facial and behavioural pain responses during painful medical procedures [41, 62]. Discrepancies between different measures of pain (e.g., behavioural versus self-report measures) are not unusual [77]; therefore it is possible that the self-report data obtained in this study represent a unique perspective on the subjective pain experience for youths with ASD. It is also possible that youths with ASD, although experiencing comparable levels of pain as typically developing children (as evidenced by the current data), express their pain in a more behaviourally and facially reactive manner (as evidenced in prior research). However, despite these issues, Bandstra et al. [74] highlighted the potential confounders that may have been present in the other major studies which did find increased facial pain response to the individuals with ASD. Specifically the study by Nader et al. [62] was confounded by its use of a bundling procedure (wrapping the child in a blanket for the purpose of constricting movement during the procedure) in preparation for the venupuncture procedure for the group of individuals with ASD and not the control group, a difference which could have accounted for the significantly greater pain responses evidenced by the children with ASD as compared with the controls in that study [62]. Also a recent study showing greater behavioural response in children with ASD also found higher levels of a physiological marker for stress in the ASD sample [41]. This indicates the possibility that the increased behavioural reactivity may not be an expression of pain; rather they are distressed at undergoing a medical procedure. Another important factor to consider in trying to understand pain in individuals with ASD is the degree of functioning of this group, which varies across the studies. For instance, Tordjman et al. [41] used a sample of nonverbal and low-functioning individuals with ASD, while Nader et al. [62] omitted any information about the level of functioning in their sample. The study by Bandstra et al. [74], on the other hand, used a high-functioning sample of children and adolescents with ASD. Also, Bandstra et al. [74] investigated pain responses in children aged 8 to 18 years, which represents a significantly older age group than the participants included in the study by Nader et al. [62]), for example, and aspects of the pain experience (e.g., ability to provide self-report) are known to change as typically developing children grow older [78]. Similar to the youth ratings, no differences emerged between the ASD and control groups for parent ratings of the amount of pain they would expect their children to show. However, this finding does not necessarily mean that youths with ASD express their pain in the same way as typically developing youths. Rather, parents of the children in this group may have grown accustomed to their children's idiosyncratic pain expressions (e.g., angry responses) over time and have learned to interpret their child's cues accurately. Although group averages for parent and child ratings were similar, additional correlations demonstrated a lack of concordance between parent and child dyads which is not a surprising phenomenon in paediatric pain assessment. This finding may even provide further support for the argument that the pattern between parent and child pain ratings is consistent regardless of whether or not the child has ASD. Furthermore, the lack of concordance in ASD is important as it indicates that, as with typically developing children, it is important for clinicians to gather pain intensity ratings from youths with ASD, rather than only relying on parent report. 

In their abstract, Daughters et al. [79] report their findings of a pilot study they carried out to examine pain and distress experienced by children with ASD during a dental cleaning procedure. The authors hypothesised that children with ASD would exhibit greater levels of behavioural distress and pain during the dental procedure compared to control children. Five children with a diagnosis of ASD and four control children participated in the study (ages 7–11 years) scheduled for a dental cleaning procedure (without sedation) took part in the pilot study. Prior to the dental cleaning procedure, caregivers were asked to complete a behavioural checklist to identify the severity of the child's stereotyped behaviours, social interaction, and communication difficulties. The dental cleaning procedures were videotaped and were later coded using a variation of the Brief Behavioral Distress Scale (BBDS), an observational measure of children's procedure-related distress. After the dental cleaning procedure, the caregivers were asked to complete the Non-Communicating Children's Pain Checklist-Revised (NCCPC) in order to assess their child's pain. The findings revealed that the mean pain scores during the dental procedure were indicative of pain for both groups. However, the children with ASD exhibited greater pain scores (*M* = 29.8) than children without ASD (*M* = 10.0). Greater levels of interfering distress behaviour were exhibited in the children with ASD compared to children without ASD. There were also moderate associations between severity of ASD symptoms and pain during the dental cleaning procedure (*r* = 0.55) and interfering distress behaviours (*r* = 0.43), with increased severity of the child's symptoms relating to higher levels of pain and distress. In sum, this pilot study indicates that individuals with ASD are more sensitive to pain during dental cleaning procedures. 

### 3.5. Embodied Pain in ASD

Observing emotions or bodily sensations in another individual produces brain activations largely overlapping those which are activated during the direct experience of the same feelings. This overlap in activated brain regions between observed and directly experienced emotions or bodily sensations indicates that empathic brain responses may rely on resonant, mirror-like systems [80–82]. The idea that empathy for pain may be mediated by mirror systems emerged with the finding that neurons in the anterior cingulate cortex (ACC) fire in response to both pain in the self and the observation of pain in another [83]. Although ASD are often described in terms of reduced empathic abilities [84], evidence for reduced empathy in domains different from mentalising and perspective taking (for instance pain) is sparse. To investigate this, Minio-Paluello et al. [85] used a sample of sixteen right-handed men with Asperger's Syndrome (a type of ASD) (aged 28.0 ± 7.2 years) and 20 neurotypical controls (aged 25.3 ± 6.7 years) age, sex, and IQ matched. 

Minio-Paluello et al. [85] used single-pulse transcranial magnetic stimulation (TMS) to explore a rudimentary form of empathy, called “sensorimotor contagion”, elicited in neurotypical participants when they observe painful stimuli applied to the body of another person. The authors regard sensorimotor contagion to have taken place when there is a reduction of corticospinal excitability recorded from the specific body part that is vicariously affected by the observed painful stimulation, in this case, the hand muscles. This inhibition to observation of pain inflicted on another body is characteristic of the corticospinal inhibition found during actual noxious stimulation (when the pain is directly inflicted). So in the study carried out by Minio-Paluello et al. [85], participants underwent single-pulse TMS during observation of painful and nonpainful stimuli affecting another individual. Motor-evoked potentials (MEPs) induced by focal single-pulse TMS of the left primary motor cortex (M1) were simultaneously recorded from two right-hand muscles, the first dorsal interosseous (FDI), and the abductor digiti minimi (ADM). Four types of video clips (each lasting 1.8 seconds) were presented on a 19-inch screen. Video clips were (1) “static”: static right hand; (2) “Pain”: needle deeply penetrating the FDI muscle; (3) “Touch”: cotton swab gently touching the FDI region; and (4) “Tomato”: needle deeply penetrating a tomato. Thus, whereas participants' FDI muscle was vicariously involved by the painful stimulation, the ADM muscle served as a somatotopic control because it was not shown to be penetrated. Previous studies of TMS show that watching moving body parts or hands increased corticospinal excitability. In order to eliminate this confounding effect, the hands in the video were static in the clips and the syringe holder was not visible. In addition to these objective neurophysiological measures, participants were also asked to imagine how the pain would feel, if applied to them. The qualities of the imagined pain were measured using the McGill Pain Questionnaire (MPQ) [86], which is made up of Sensory (items 1–10, 17–19) and Affective (items 11–15, 20) subscales, and through the Hurts value, a rating between 0 and 10 indicating how much the participants thought the injection would hurt them. 

 Minio-Paluello et al. [85] found that when observing other's pain, participants with ASD, in contrast to neurotypical control participants, did not show any amplitude reduction of motor-evoked potentials recorded from the muscle vicariously affected by pain, nor did their neurophysiological response correlate with imagined pain sensory qualities. All experimental video clips were similarly rated by the two groups (*ps* > 0.10) except for the Static condition, which was significantly less arousing for ASD (*P* < 0.02). Participants with ASD, compared with control participants, perceived themselves less able to identify with the model being touched (*t* = 2.07, *P* = 0.050) and tended to judge the touch as more painful (*t* = −1.82, *P* = 0.08). When asked to imagine how they would feel if receiving the painful stimulation shown in the videos and to rate the sensory and affective qualities of imagined pain, control participants and individuals with ASD gave similar ratings (all *ps* > 0.33). Therefore the lack of sensorimotor contagion in ASD cannot be explained by group differences in the imagined “painfulness” of the observed events. They were therefore able to correctly understand or identify how painful a particular event would be despite showing abnormal neurophysiological responses. Although participants with ASD did not embody others' pain, the observation of painful stimuli inflicted to the hand muscle of another person inhibited control participants' corticospinal representation of the same muscle (i.e., the FDI muscle). Those MEPs recorded from the ADM muscle are not modulated cannot be explained in the terms of reduced reactivity of this muscle. Indeed, when videos depict the ADM being penetrated by a needle, similar corticospinal inhibition of this muscle has been observed [87]. In sum, finding no embodiment of others' pain or reduced empathic abilities in individuals with ASD (as evidenced by reduced or absent sensorimotor contagion during the observation of pain affecting another person, the hand in the movie clips) provides neurophysiological evidence for reduced empathic resonance in people with ASD and suggests that their difficulties with empathy is mediated not only by cognitive dimensions but also by sensorimotor resonance with others. 

### 3.6. Relationship between Opioid Hormone and ASD

Nagamitsu et al. [88] measured cerebralspinal fluid (CSF) levels of beta-endorphin, an opioid hormone, in 19 Japanese children (17 boys, 2 girls, mean age 4.23 years, range 2.00–6.42) with typical infantile autism (ASD). Some children presented with accessory symptoms such as self-injurious behaviour (3/19), pain insensitivity (8/19), and stereotyped movements (10/19). The controls consisted of 23 age-matched Japanese children (18 boys, 5 girls, mean age 3.78 years, range 0–10.75) who had undergone lumbar puncture for the diagnosis of a possible central nervous system (CNS) infection but whose CSF showed normal results. CSF levels of p-endorphin in three patients with the Rett syndrome (3 girls, ages 10–14 years) who presented with symptoms resembling those of infantile ASD were also recorded. In infantile autism, CSF levels of beta-endorphin did not differ significantly from those of age-matched controls. No significant correlation between CSF levels and clinical symptoms, including self-injurious behaviour, pain insensitivity, and stereotyped movement was found. However, CSF beta-endorphin levels were significantly higher in the patients with Rett syndrome than in the control (*P* < 0.05). Findings indicated that neurons containing beta-endorphin may not be involved in patients with infantile autism, therefore not supporting the relationship between dysfunction of brain opioid and ASD.

### 3.7. Oversensitivity to Pain and Age of Diagnosis of ASD

Mandell et al. [89] attempted to identify factors which may delay diagnosis of ASD among a community sample of children with ASD. Survey data was collected in Pennsylvania from 969 caregivers of children who had ASD and were younger than 21 years regarding their service experiences. The average age of diagnosis was 3.1 years for children with autistic disorder, 3.9 years for pervasive developmental disorder not otherwise specified, and 7.2 years for Asperger's disorder (a type of ASD). Interestingly, oversensitivity to pain was associated with a 0.6-year increase in the age of diagnosis. The association of oversensitivity to pain with later diagnosis may be because this symptom prompts clinicians to search for other organic causes and not consider developmental issues.

### 3.8. Level of Functioning in ASD Group across Case and Experimental Studies

Of the five case studies only one specifies level of functioning and was mild ASD [17]. The remaining four case studies do not indicate level of functioning [32, 33, 56, 58]. Of the ten experimental studies, two studies include high functioning individuals with ASD [72, 74]. Three studies did not specify level of functioning [70, 79, 88]. One did not specify low or high functioning using clinical guidelines and examined a variety of ASD symptoms to determine a level of functioning but does not report how many are contained within each category [89]. Another study, rather than defining in terms of high and low functioning, describes levels of severity based on scores on the Autism Spectrum Quotient (AQ, [90]) [85]. Two studies used the same data and employed The Childhood Autism Rating Scale (CARS, [91]) to create two groups: severely autistic and mildly-moderately autistic and the majority of the group fell into the severely autistic range [46, 62]. Lastly, another study had 39 patients in the severe ASD group and 39 in the “mild” to “moderate” ASD group, assessing ASD severity using the Autism Diagnostic Interview-Revised (ADI-R, [92]). [41]. Four of the experimental studies did not include a comparison/control group [46, 70, 79, 89]. 

## 4. Discussion

Five case studies and ten experimental studies were found in the PRISMA search which investigated some aspect of pain in individuals with ASD. All five case studies described individuals with ASD who were exhibiting pain *insensitivity* [17, 32, 33, 56, 58]. The two cases presented by Bursch et al. [33] demonstrate how chronic pain can be the focal symptom and perseverative focus of attention for individuals with an ASD. Once focused on pain, difficulties in shifting attentional focus can serve to increase pain and associated distress. Therefore, implementing a treatment that somehow interrupts the perseveration might reduce or even eliminate the pain that the individual experiences. In fact, this is exactly what Zeltzer and Schlank [93] found. They describe a case in their pain clinic where a child with ASD presented to them yelling repeatedly, “Ow! Ow! Ow!” Two months earlier, the child had sustained a leg injury and he had been shouting ever since. Zeltzer and Schlank suggested that rather than shouting, the child should replace this by squeezing a ball instead. Interestingly, this resulted in a transfer of his expression to the point where he actually reported feeling better because he no longer felt embarrassed about repeatedly shouting “Ow!”. These findings strongly indicate that treating the perseveration can be the most effective way to reduce suffering for some patients [7]. What has also been proposed by numerous researchers is that in children with ASD the anxiety often experienced by this population might actually contribute to their pain experience. This holds especially true for those individuals whose muscles remain tense for extensive durations [93].

Also important for clinical practice is the case study by Mieres et al. [32] which suggests a particular approach is required in assessing the subjective feeling of pain in individuals with ASD. Rather than ask how much pain they are feeling, they suggest instead a new series of questions, the key being “How uncomfortable are you?” (used in addition to the verbal and pictorial pain scale). 

Of the ten experimental studies only one found no significant difference in pain sensitivity between patients with ASD and controls. Specifically, no significant differences were found between the pain ratings of youth with ASD or their parents as compared with a sample of typically developing youths [74]. Interestingly, overall, only one of the experimental studies (compared to all five of the case studies) found evidence of underreactivity to pain (suggestive of pain insensitivity) in 40% of their sample [70]. One study [41] found that individuals with ASD do not have a decreased sensitivity for pain and investigated both behavioural reactivity to pain during venepuncture as well as plasma b-endorphin concentrations and heart rate. The additional physiological measures were particularly important since there was an absence of any behavioural pain reactivity in the individuals with ASD during venepuncture despite their higher heart rate and plasma b-endorphin levels—strongly suggesting that the individuals with ASD were not insensitive to pain.

Five studies found evidence of a greater degree of pain sensitivity in individuals with ASD [62, 72, 79, 85, 89]. Interestingly, oversensitivity to pain was associated with a 0.6-year increase in the age of diagnosis [89]. One study [46] investigated the influence of information about the pain experience of children with ASD on observers' judgement of pain intensity in children with ASD and examined the impact of facial activity on observers' judgement of pain intensity in children with ASD. Facial activity was found to have a significant impact on observers' estimates of pain intensity; pain sensitivity information did not [46]. This is in contrast to the view that parents' ratings of pain in their children with ASD may be distorted due to misinformation about pain insensitivity in their children [62]. A possible limitation with this study, in terms of investigating the effect of information of pain sensitivity in individuals with ASD, is that the students in this study had no personal relationship to the individuals they observed and rated in the videos. This might have contributed to the lack of effect of pain information on the judgments made. A different picture might emerge if the same situation was applied to individuals with a personal relationship to the child such as a parent. These results have important implications for the assessment and management of pain in children with ASD [46]. The finding that observers may be able to decode pain information from facial activity [46] is important because children with ASD frequently lack the skills to express their pain verbally and this could put them at risk for substandard health care. The findings of a significant behavioural reaction in response to a painful stimulus in individuals with ASD [62, 72, 79, 85, 89] contradict the widespread belief in the literature of pain insensitivity in individuals with ASD. Some of the findings reported in this review also question the clinical appropriateness of parental global report as an assessment tool for pain in children with ASD [62]. Another study investigated whether P-endorphin plays an important role in infantile autism by measuring the cerebrospinal fluid (CSF) levels of p-endorphin and evaluated the correlation between these levels and ASD symptoms [88]. Findings did not support the opioid hypothesis to explain pain sensitivity in ASD [88]. 

 Another important aspect is whether the five experimental studies that included different levels of functioning or severity of ASD [41, 46, 62, 85, 89] report whether this had any notable effect on the results. For instance, were the findings weaker for individuals who are higher functioning. Two studies did find differences [41, 85] while three did not [46, 62, 89]. Tordjman et al. [41] found that plasma b-endorphin levels were positively associated with ASD severity. Minio-Paluello et al. [85] found that corticospinal inhibition was maximal in the individuals with fewer ASD traits. 

### 4.1. Expression of Pain in Individuals with ASD

Numerous anecdotal reports show that caregivers frequently describe unusual, or absent, responses to painful stimuli in their children with ASD. Some caregivers are even able to describe unique behaviours in their child that enable them to know when they are in pain. However, it is crucial to point out here that altered pain expression is not universally observed in ASD. Despite this most experts are in agreement that the pain experience appears different in individuals with ASD [94]. 

Tordjman et al. [41] argue that their findings indicate that prior reports of reduced pain sensitivity in ASD are related, not to an insensitivity or endogenous analgesia to pain but to a different mode of pain *expression. *This is without doubt the most crucial finding and clearly further investigation to explore this aspect is required. The findings by Tordjman et al. [41] constitute a clear challenge to theories of reduced pain sensitivity in ASD since they found that painful stimuli can produce physical and psychic stress in individuals with ASD and that this stress can be manifested by physiological responses and expressed through autistic behaviours. Tordjman et al. [41] hypothesise that the different mode of pain expression in individuals with ASD may be mediated by (1) verbal communication impairments, (2) deficits in non-verbal communication and body image problems (difficulty locating the painful area), or (3) other cognitive problems such as (a) difficulty in establishing cause-effect relationships between the pain sensation and the stimulus causing the pain, (b) problems discriminating, representing and identifying sensations and emotions which involves abstraction and symbolisation capacities (the perception of pain integrates sensorial, emotional, and cognitive factors [95]), (c) problems of learning socially appropriate responses to pain [41]. 

The majority of the experimental studies included in the review examine pain reaction to a specific medical procedure venepuncture. Therefore, the findings cannot be generalised to other contexts and pain situations such as pain during everyday situations and experience of chronic pain in individuals with ASD. It is also important to acknowledge that the experiences of children with ASD occur along a spectrum of severity. Therefore it is highly possible that the experience and expression of pain may differ depending on where the individual lies on this spectrum (Messmer et al. [46]); the level of communicative and language abilities of individuals [94] and the impact of different ASD diagnoses (i.e., Asperger's disorder; PDD-NOS; Rett syndrome) on pain expression and reactivity. This variability again emphasises that interventions and treatments must be tailored to each specific child [94].

Despite the complexity in interpreting the findings from the sparse amount of studies to date which have looked at pain sensitivity in individuals with ASD, what is clear is that there is a need for a pain assessment tool specifically for use in this population [94]. Existing instruments may be inappropriate. To my knowledge, there has only been one study (from a dissertation submitted to the University of Florida for the degree of Doctor of Philosophy) which has attempted to identify whether there are unique pain indicators applicable to a significant amount of children with ASD and whether there are pain indicators identified by caregivers that are completely unique to the one child. Inglese [94] found several objective, observable and measurable indicators which were presumed by the author to be relevant to pain assessment in ASD (i.e., “furrowed brow,” “banging his/her head,” “injuring oneself,” “grimacing,” “guarding,” and “increased heart rate”), many other identified indicators were subjective and required (a) knowledge of the child's baseline, and (b) monitoring for changes from normal (i.e., “crankiness,” “being less active,” “rocking unusually,” “acting “off”, and “irritability”). Inglese [94] suggests then that in the designing of a new instrument specifically for pain assessment in individuals with ASD, there needs to be the inclusion of sections which are objective and quantifiable—these could be used by individual(s) who are unfamiliar to the child being assessed—in addition to sections which are based on caregiver judgments regarding what is typical in their child. In effect encompassing both caregiver information regarding their child's baseline and objective assessment questions which can be conducted by individuals unfamiliar with the child is crucially important in creating a comprehensive pain assessment in this population [94].

### 4.2. Limitations

Sensations are often thought to be logically private, subjective, self-intimating, and the source of incorrigible knowledge for those who have them. Since pain is often thought to be a “subjective” experience, this has lead researchers to use the report as the gold standard for pain experience. Many of the studies identified in the present review investigate pain reactivity using caregiver reports which have obvious limitations such as reporting bias. However, the most notable limitation with this is highlighted by research which suggests that parents of children with ASD perform worse than parents of control children in facial emotion task. For instance, in an emotional labelling task in response to schematic facial patterns representing five basic emotions, parents of children with ASD performed worse than parents of control children (i.e., [96]). The study by Nader et al. [62] (one of the studies reported in this review) is consistent with the difficulties found in parents of children with ASD in correctly interpreting facial emotions. Nader et al. [62] found that some of the caregivers did not interpret their child's pain expression accurately in that children with ASD who had been assessed by their parents as having a lower pain sensitivity and reactivity tended to show greater facial reactions and behavioural distress in response to the venepuncture.

Similarly, there is the issue of asking individuals with ASD to rate pain according to facial expressions of pain (i.e., Faces Pain Scale (FPS)). Numerous researchers maintain that individuals with ASD have difficulty with processing facial expression (i.e., [97]). Individuals with ASD process faces differently and show reduced attention to faces and facial expressions [98]. This reduced interest in faces is likely to impair their face processing skills, so that children with ASD do not become “face experts” like their typically developing peers [99].

The majority of the small number of studies which investigated some aspects of pain specifically in individuals with ASD are limited by their sample size. Seven of the ten experimental studies had sample sizes of ASD groups less than 21. The remaining three had 73, 969, and 208 individuals with ASD. More studies with larger sample sizes examining behavioural reactivity to pain as well as measuring physiological responses are imperative to our understanding of pain experience in individuals with ASD. A further complication in attempting to draw conclusions from the literature to date are the differences across studies in terms of cognitive development of the individuals with ASD (not to mention the wide differences in age). For instance, in one study [41] all patients were cognitively impaired: mean full scale IQ = 42.2, SD = 3.2 (range 40–58) while another study all had IQ of at least 70 [72]. These limitations and differences pose a significant problem when trying to determine whether there is pain insensitivity, greater pain sensitivity or no difference in individuals with ASD. 

Lastly, it is also difficult to disentangle from the experimental studies conducted to date as to how much of the observed pain reactions, and so forth were due to the individuals with ASD level of *distress* rather than any pain response. Individuals with ASD might display more distress during the venepuncture procedure compared to controls and this might be completely independent from how painful they find the experience. 

### 4.3. Future Directions and Clinical Implications

Further studies are needed to recognise illnesses earlier in the absence of pain or pain perception in children with an ASD and to develop reliable and valid metrics for pain identification for both verbal and nonverbal individuals with ASD [32]. Further research is also needed to explore the concordance between parent report of pain sensitivity and observed reactivity of children with ASD to every day painful incidents. Children with ASD may exhibit typical behavioural reactions to procedural pain in a clinical setting but atypical responses to everyday pain in their home environment [62]. It is important to understand how different types of pain in different settings are perceived in order to acceptably manage pain in children with ASD. Additionally, more research is needed to understand how observers decode the pain experience of children with ASD and explore the potentially biasing effect of pain sensitivity information on observers' estimates of pain in children with ASD [46]. The study by Mieres et al. [32] also stresses the importance of the way in which clinicians ask patients with ASD about how much pain they are experiencing. They found that asking the individual with ASD how uncomfortable they were on a scale of 1 to 10 was a more accurate representation of the pain they felt than when they were asked directly how much pain they were in on a scale of 1 to 10. 

Further research is also need to explore further how children and adults with ASD react to various types (acute versus chronic) or degrees of pain. The need for this research is emphasised by the study reported in this review [41] that a significant number of individuals with ASD reported absent or reduced pain reactivity (41.3% versus 8.7% of controls) but in the same sample the majority (78%) exhibited normal pain reactivity to burning based on caregiver report. This clearly shows that the type and severity of the pain event is important when studying pain in this population. 

In a study just published, Wager et al. [100], based on four studies involving a total of 114 participants, developed an fMRI-based measure that predicts pain intensity at the level of the individual person and found that it is possible to use fMRI to assess pain elicited by noxious heat in healthy persons. Given the limitations of the experimental studies to date (for instance the reliability of self-report measures of pain and caregiver report of pain in their child), this would be an more robust and objective way to investigate whether pain experience is any different in individuals with ASD compared to controls. This might also have the potential to show whether there is a discordance between neural signatures of pain and the expression of pain in the individual with ASD.

## 5. Conclusions

There is still relatively little research on the unique problems posed by the expression of pain and sensitivity to painful stimuli in individuals with intellectual and developmental disabilities [101] and particularly in individuals with ASD from childhood to adulthood. Overall, the findings reported here of a significant behavioural reaction in response to a painful stimulus in individuals with ASD contradict the wide spread belief in the literature of pain insensitivity in individuals with ASD. The case studies all reported pain insensitivity in individuals with ASD and provide an example of how impaired sensory perceptions can mask and delay the ability of health care professionals to recognise the need for treatment. However, the majority of the ten experimental studies reviewed here indicate that the idea that individuals with ASD are pain insensitive needs to be challenged. This systematic review highlights the need for a shift away from the widely and long-held belief that children and adults with ASD have a reduced pain sensitivity, do not feel pain as intensely as others, and have an indifference to pain and a high threshold for pain. What was also highlighted by some of the findings of this review is the importance of further study to explore the theory that the pain *expression* in individuals with ASD differs from that of neurotypicals. Recognition of all these findings have important implications for the treatment and recognition of the need for treatment in individuals with ASD.

## Figures and Tables

**Table 1:** Case report studies which explored pain perception, expression, or observer perception of pain in individuals with ASD.

Author	Samples	Level of functioning of ASD sample	Aim of the study	Findings
Bursch et al. [33]	2 patients with the signs and symptoms of ASD.	Does not specify.	Case report of 2 patients and their sensory abnormalities and pain perception as observed by family.	Reported evidence of lack of pain sensitivity in both patients. That is, “…once grabbed a hot frying pan and did not seem to respond in a way typical of someone in pain.”

Elwin et al. [56]	17 works published in English or Swedish and 10 autobiographies.	Does not specify for individuals.	To explore hyper- and hypo-sensitivity in individuals with ASD in the context of verbal expression. Using samples of published autobiographies as a data source.	Yes, self-reported pain insensitivity. Pain could be indistinctly experienced, and several authors pointed out having a very high pain threshold. Injuries could easily go undetected.

Mieres et al. [32]	9 year old with pervasive developmental disorder, not otherwise specified.	Does not specify.	To describe how a nurse and a physical therapist in an interprofessional (IP) school-based clinic collaborated to meet the needs of a child with PDD-NOS, with atypical classroom behaviours and declining student performance.	Yes: parental report of pain insensitivity. For this student, pain was an unreliable indicator of both a dental infection and piercing of skin by thorny objects. Child was referred to a dentist who discovered a severe abscess affecting two teeth. The dentist reported that an abscess of this size without pain is unusual, given the size, the depth, and the proximity to bone.

Ross-Russell and Sloan [17]	7-year-old boy.	Mild ASD.	Report of a case of a young child with mild ASD who presented with unexplained dental pain and who subsequently went on to extract his own lower right deciduous canine tooth.	Yes, suggestion that this patient had pain insensitivity. “He has also demonstrated SIB in the form of head banging. He was brought to the clinic complaining of pain of about one week's duration that was increasing in intensity which was present most of the time and was not sensitive to hot, cold or sweet stimulus. Within 24 hours the patient was again back at the clinic, as the pain had not resolved with ibuprofen, and by this time his lower right deciduous canine was very slightly mobile. No evidence of alveolar bone loss and the tooth had normal root anatomy. By the next day the patient had extracted his lower right deciduous canine tooth, witnessed by his mother. He claimed it had been ‘itching' him until he got it out.”

Rutherford [58]	Twins: boy who was diagnosed with ASD at 3 years and 1 month, and a girl who developed typically.	Does not specify.	Describes the development of an infant who was later diagnosed with ASD. Directly compares his development to that of his twin from a prenatal period through to the age of 4 years. Explored through examination of personal journals and medical records kept by the mother.	Yes: evidence of pain insensitivity—maternal observations. N.F. frequently showed insensitivity to pain which was exhibited as early as 6 to 12 months of age.

**Table 2:** Experimental studies which explored pain perception, expression, or caregiver/observer perception of pain in individuals with ASD.

Author	Samples	Level of functioning of ASD sample	Aim of the study	Findings
Bandstra et al. [74]	20 ASD (17 boys; 3 girls) & 20 TD controls (16 boys; 4 girls). Age range: 8–18 years.	High	Assessing self-report of pain using vignettes and also comparing this to parental reports.	No significant differences in pain intensity ratings between the ASD and controls. No significant differences between the pain ratings of youths with ASD or their parents as compared with a sample of typically developing youths.

Cascio et al. [72]	8 adults with ASD (clinical diagnoses of either Autistic or Asperger Disorder; all had IQ of at least 70 (7 males & 1 female (mean age 29.3 years, range 20–45). 8 adults without ASD (sex and age matched). (mean age 29.0, range 21–45).	High	To investigate tactile sensitivity in adults with autism using a variety of stimuli, in order to probe different submodalities of somatosensation. Experiment specifically related to present review—thermal pain threshold.	Yes, significant difference—ASD group showed a greater degree of pain sensitivity. For cold pain, there was a main effect of site (*F* = 5.12, *P* = 0.0250), and group (*F* = 3.84, *P* = 0.0518), with the ASD group displaying average cold pain thresholds of 16.68°C, compared to the control group average of 9.04°C. For heat pain, there was a significant effect of group. The average threshold for the ASD group was 43.66°C, while that of the control group was 46.58°C.

Daughters et al. [79]	5 children with a documented ASD (between the age 7–11 years).	Does not specify.	A pilot study to investigate pain and distress in children with autism during a dental cleaning procedure.	Children with ASD exhibited greater pain scores (M = 29.8) than children without ASD (M = 10.0). Greater levels of interfering distress behaviour were exhibited in the children with ASD. Moderate associations between severity of ASD symptoms and pain during the dental cleaning procedure (*r* = .55) and interfering distress behaviours (*r* = .43), with increased severity of the child's symptoms relating to higher levels of pain and distress.

Klintwall et al. [70]	Population-based group of 208 20–54-month-old children, diagnosed with ASD and referred to a specialised habilitation centre for early intervention. Children were sub-grouped (8 in total) based upon degree of ASD symptoms & cognitive level.	Subgroups (i.e., classic autism, nuclear autism)—but does not specify low or high functioning.	To describe sensory abnormalities in preschool children with an ASD, compared to different subgroups within the autism spectrum in terms of the presence of sensory abnormalities, and relate the findings to other clinically relevant symptom domains.	Yes: significant differences in pain sensitivity. Under-reactivity to pain in 40% of the sample. Under-reactivity to cold and heat were reported for 22% and 7%, respectively. Children with self-injurious behaviours had more sensory abnormalities affected (M = 2.0, SD = 1.5, *n* = 61) than children with no such behaviours (M = 1.3, SD = 1.2, *n* = 147); *t*(206) = 2.791, *P* = 0.006.

Mandell et al. [89]	Survey data were collected in Pennsylvania from 969 caregivers of children who had ASD and were younger than 21 years regarding their service experiences.	Does not specify low or high functioning in terms of DSM and so forth.	Early diagnosis of children with ASD is critical but often delayed until school age. This study attempted to identify these factors among a community sample of children with ASD.	Oversensitivity to pain was associated with a 0.6-year increase in the age of diagnosis.

Messmer et al. [46]	6 ASD individuals (4 boys and 2 girls between 3 and 7 years).	Video clips of children with ASD undergoing venepuncture were obtained from a previous study [62]; see below for details.	To examine the influence of information about the pain experience of children with autism on observers' judgement of pain intensity in children with ASD and to examine the impact of facial activity on observers' judgement of pain intensity in children with ASD.	Facial activity had a significant impact on observers' estimates of pain intensity while pain sensitivity information did not.

Minio-Paluello et al. [85]	16 right-handed men with ASD (aged 28.0 ± 7.2 years) and 20 neurotypical controls (aged 25.3 ± 6.7 years) age, sex, and IQ matched.	Mention levels of severity in terms of score on the AQ.	To explore whether people with AS differ from neurotypical control participants in their empathic corticospinal response to the observation of others' pain and the modulatory role played by phenomenal experience of observed pain and personality traits.	Participants with AS, compared with control participants, tended to judge the touch as more painful (*t* = −1.82, *P* = 0.08).

Nader et al. [62]	21 3-year-old to 7-year-old children with ASD and 22 nonimpaired children.	Mean CARS score for the ASD group was 39.10 (SD = 4.98, range 30.5–47), which put the average for the group into the severely autistic range (CARS score >37). 9 fell into the mildly-moderately ASD range (CARS score 30–37), and 12 fell into the severely ASD range.	Aims of the study were to (1) characterise the behavioural response of children with ASD experiencing a venepuncture using objective observational measures of pain and distress, (2) examine parents' assessments of pain behaviour in children with and without autism, including comparison of the relationship of parental reports with behavioural measures, and (3) compare the behavioural reactions and parental assessments of children with ASD with children without ASD undergoing venepuncture.	In contrast with many of the other studies reported in this review, this study found evidence which indicates that individuals with ASD do not have an insensitivity to pain as manifested by a lack of behavioural response—children with ASD display a significant behavioural reaction in response to a painful stimulus. Using FPS scores as a measure of parental assessment of pain response following the venepuncture, parents of children with ASD reported observing more pain in their children during the venepuncture (M = 4.29, SD = 1.45) compared with parents of the children without autism (M = 2.75, SD = 1.90; *t*(41) = 2.97, *P* < 0.05). Using the NCCPC as a retrospective measure of parental assessment of typical pain reactivity in their children, scores did not differ between the autism group (M = 60.33, SD = 13.50) and comparison group (M = 58.41, SD = 14.19; *t*(41) = 0.46, *P* > 0.05). Parent reports of pain temperament in children with ASD (M = 2.72, SD = 1.32) were similar to parent reports of pain temperament in the children without ASD (M = 2.82, SD = 1.30; *t*(38) = −0.23, *P* > 0.05).

Nagamitsu et al. [88]	19 Japanese children (17 boys, 2 girls, mean age 4.23 ± 1.18 years, range 2.00–6.42) with typical infantile autism. 23 controls—age-matched Japanese children (18 boys, 5 girls, mean age 3.78 ± 3.37 years, range 0–10.75). 3 patients with Rett syndrome (3 girls, ages 10–14 years).	Does not specify.	To clarify whether P-endorphin plays an important role in infantile autism, we determined the cerebrospinal fluid (CSF) levels of p-endorphin and evaluated the correlation between these levels and ASD symptoms.	Finding do not support the opioid hypothesis to explain pain sensitivity in ASD. No significant correlation between CSF levels and clinical symptoms, including self-injurious behaviour, pain insensitivity, and stereotyped movement. However, CSF levels of p-endorphin were significantly higher in the patients with Rett syndrome than in the control (*P* < 0.05). Data suggest that neurons containing p-endorphin may not be involved in patients with infantile autism.

Tordjman et al. [41]	73 children and adolescents with ASD and 115 control matched for age, sex and pubertal stage. (ASD group 49 males and 24 females. ASD group total age 11.7 plus or minus 4.5; comparison group 75 males and 40 females. Total age 12.7 + or −5.9).	Individuals with ‘‘severe” ASD (*n* = 39). Individuals with ‘‘mild” to ‘‘moderate” ASD (*n* = 39). Normal controls (*n* = 103).	To examine behavioural and physiological pain responses, plasma b-endorphin levels, and their relationship in a large group of individuals with ASD.	No: individuals with ASD do not have decreased sensitivity to pain. A high proportion of individuals with ASD displayed absent or reduced behavioural pain reactivity at home (68.6%), at day care (34.2%), and during venepuncture (55.6%). Despite their high rate of absent behavioural pain reactivity during venepuncture (41.3 versus 8.7% of controls, *P* < 0.0001), individuals with ASD displayed a significantly increased heart rate in response to venepuncture (*P* < 0.05) which was significantly greater than for controls (mean 6 SEM; 6.462.5 versus 1.360.8 beats/min, *P* < 0.05). Plasma b-endorphin levels were higher in the ASD group (*P* < 0.001) and were positively associated with ASD severity (*P* < 0.001) and heart rate before or after venepuncture (*P* < 0.05), but not with behavioural pain reactivity.

Key:

AQ: Autism Spectrum Quotient [90].

CARS: The Childhood Autism Rating Scale [92].

CFS: Cerebralspinal fluid.

CNS: Central Nervous System.

PDD-NOS: Pervasive Developmental Disorder Not-Otherwise Specified.

TD: Typically developing.
